# Synergistic effect of hypocrellin B and curcumin on photodynamic inactivation of *Staphylococcus aureus*

**DOI:** 10.1111/1751-7915.13734

**Published:** 2021-01-19

**Authors:** Yali Li, Yi Xu, Qiaoming Liao, Mengmeng Xie, Han Tao, Hui‐Li Wang

**Affiliations:** ^1^ School of Food and Bioengineering Hefei University of Technology Hefei 230009 China; ^2^ Engineering Research Center of Bio‐process Ministry of Education Hefei University of Technology 193 Tunxi Road Hefei 230009 China

## Abstract

Antimicrobial photodynamic inactivation (aPDI) serves as a new approach to control the growth of foodborne bacteria. It remains elusive if the photodynamic efficacy of hypocrellin B (HB) can be potentiated by joint action with curcumin. In this study, we measured the survival rate of *Staphylococcus aureus* strains under the varying photodynamic conditions. According to our data, a maximum of 5–6 log_10_ decrease of bacterial survival can be achieved under the tested conditions (500 nM, 9 J cm^‒2^). Regarding the bactericidal mechanisms, HB‐based aPDI disrupted the membrane integrity of staphylococcal cells, probably owing to the stimulated reactive oxygen species (ROS). In addition, aPDI disrupted the enzymatic activities of bacterial antioxidant proteins and caused the leakage of multiple intracellular substances. The HB‐mediated photodynamic efficacy was potentiated by the addition of curcumin with a sublethal dose. This dual‐photon synergy arose from unique aPDI conditions (100 nM each and 9 J cm^‒2^). The synergistic action might be accounted for by the increased type I/type II ratio of ROS, as evidenced by the effect of different quenchers. Finally, the joint use of photosensitizers reduced the microbial contamination of the tested apple while maintaining its quality. In summary, photodynamic inactivation based on dual photons showed synergistic activity in controlling the growth of *Staphylococcal aureus*, which provided a novel approach to maintain food safety.

## Graphical abstract

Photodynamic activity of HB against *S. aureus* was comprehensively profiled, and this effect can be strengthened by joint action with curcumin. HB/curcumin photo‐inactivated *S*. *aureus* through disrupting cell envelope integrity and inducing diverse set of oxidative stress, forming a synergistic aPDI method depending on the irradiated conditions.

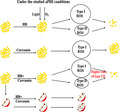

## Introduction

Food safety continues to be a major concern for public health, consumers and food industries throughout the world (Penha, *et al*., 2017; Correa, *et al*., 2020). And this serious challenge was known to arise from prevalence of microbial contamination mediated by foodborne pathogens. Among them, *Staphylococcus aureus* (*S. aureus*, SA) is one of the most prevalent Gram‐positive bacteria (Chao, *et al*., 2015). *S. aureus* strains are able to produce seven toxins responsible for multiple foodborne illnesses (Tenderis, *et al*., 2020). Due to its invasiveness and taking advantage of human immune weaknesses, increasing outbreaks of staphylococcal food poisoning (SFP) occurred pervasively, causing a broad range of disease syndromes, including life‐threatening endocarditis, meningitidis and pneumonia (Nakonieczna, *et al*., 2010; Hennekinne, *et al*., 2012). Given the circumstances, attempts should be made to control the growth and contamination of *Staphylococcus aureus* on ordinary foods.

The conventional heat‐ and chemical‐based bactericidal methods in the food industry are accompanied with some unresolved issues, such as incomplete microbial inactivation, pollution, secondary food contamination and antibiotic resistance (Silva, *et al*., 2018, Yuan and Yuk, 2018) To address it, a new technique, namely antimicrobial photodynamic inactivation (aPDI), was recently introduced for food decontamination (Asok, *et al*., 2012; Glueck, *et al*., 2017; Correa, *et al*., 2020). aPDI is defined as the application of a non‐toxic dye known as photosensitizer (PS), which can be photoactivated with light of the appropriate wavelength in the presence of oxygen, to generate cytotoxic reactive oxygen species (ROS) such as free radicals (type I reaction) and/or singlet oxygen (type II reaction) (Kashef and Hamblin, 2017; Yang, *et al*., 2019). Photodynamic approach has been developed as an efficient anticancer therapy (Hamblin, 2016), whereas its use in food microbial inactivation is still in its infancy.

The advantages of aPDI are that no toxic chemicals are generated, and there is a low possibility of triggering bacterial resistance, allowing the inactivation of a broad‐spectrum of microorganisms (Bartolomeu, *et al*., 2016; Penha, *et al*., 2017). For food preservation, the control of foodborne pathogens can be achieved by visible light, avoiding the use of harmful ultraviolet rays. In addition, given that the bactericidal capacity of aPDI is dependent on the combined use of light, oxygen and, vitally, a photosensitizer, using natural products as photosensitizers are best suited for food applications.

Hypocrellin, a natural pigment isolated from the traditional Chinese medicinal fungi *Hyprocrella bambusae* and *Shiraia bambusae*, has gained considerable attention due to its excellent light‐induced anticancer and antiviral activities (Su, *et al*., 2011; Natesan, *et al*., 2017; Qi, *et al*., 2019). Hypocrellin consists of hypocrellin A (HA) and hypocrellin B (HB), which are similar in structure, with a difference of only one hydroxyl group (Jan, *et al*., 2019). Compared to the conventional PS agent Photofrin II, HB possessed several advantages: easy preparation and purification, higher photodynamic efficiency, low dark toxicity, as well as natural origin (Xu, *et al*., 2002; Jiang, *et al*., 2014; Jan, *et al*., 2019). There are emerging examples of aPDI application based on HB: Jiang et al. found that HB‐mediated aPDI inhibited the growth of microbial cells and damaged its envelope structure (Jiang, *et al*., 2013; Jiang, *et al*., 2014). Still, understandings are lacking with respect to its comprehensive bactericidal conditions, as well as synergistic effect with other photosensitizers.

Photosensitizers were often used with other substances, mostly nanoparticle (NP) materials like TiO_2_‐NP, to enhance its photodynamic potency, increase its water solubility and extend photoresponse of TiO_2_‐NP to visible light spectrum (Xu, *et al*., 2002; Lin, *et al*., 2012; Hou, *et al*., 2016). This photodynamic action induced by dual photosensitizers showed an enhanced ROS yields. Curcumin is a natural plant pigment known for its diverse biological activities (Correa, *et al*., 2020), which was also introduced as a promising PS to elicit photodynamic reactions (Chignell, *et al*., 1994). As the absorbance peak wavelength of curcumin was in proximity to HB, it is tempting to speculate that curcumin might be able to potentiate the HB‐mediated aPDI activity, a novel dual‐PS system that is not yet established and tested.

Given it, the HB‐mediated aPDI against staphylococcal strains, as well as its causative mechanisms, was comprehensively investigated in this study. Subsequently, the synergistic effect of HB/curcumin, as well as their application on food decontamination, was further explored. This work might shed light on the natural dual‐photosensitizer‐mediated aPDI system on foodborne microbes.

## Results

### Profiling aPDI of HB on Staphylococcus aureus

**Fig. 1:**
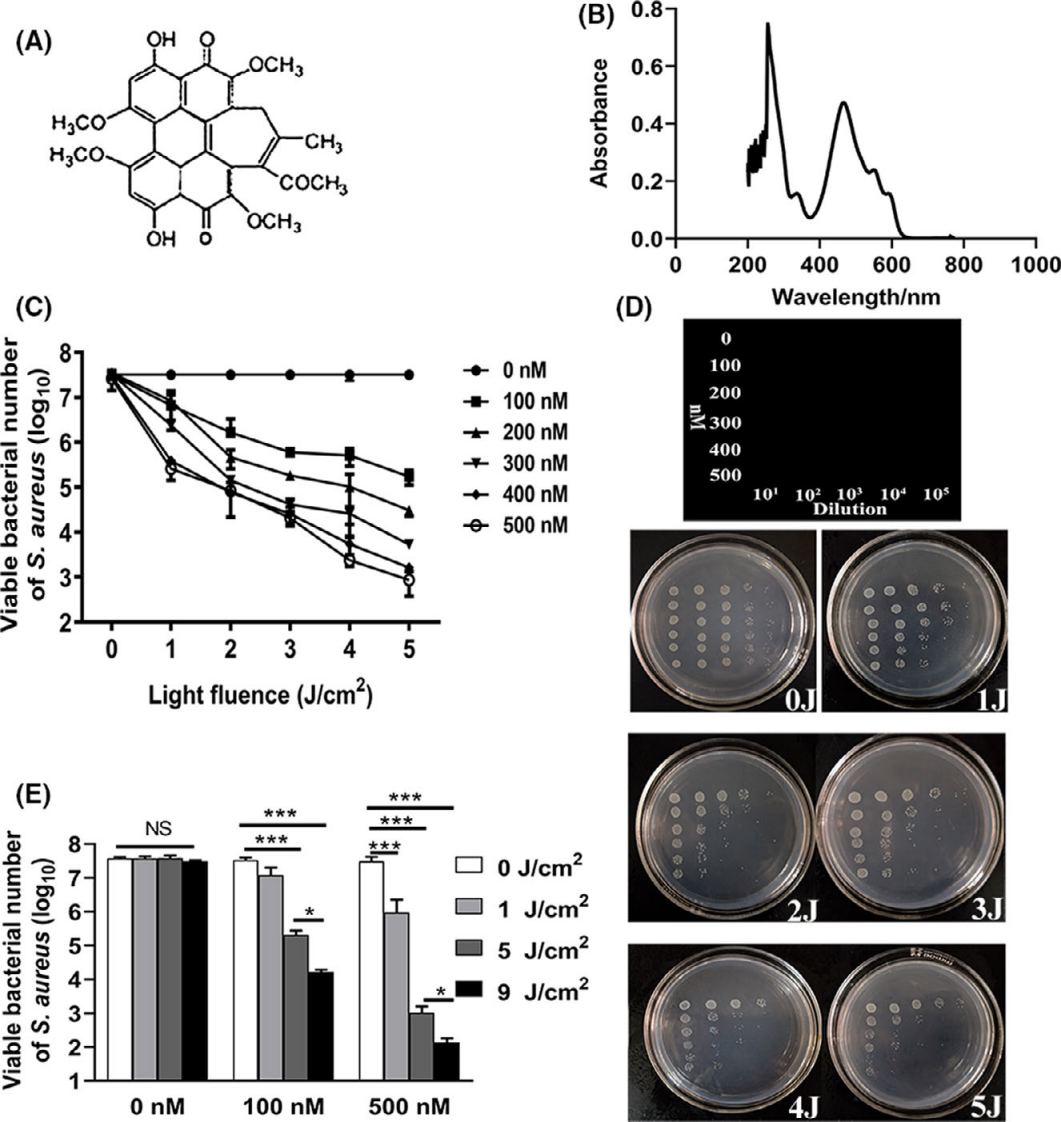
HB‐mediated photoinactivation of *S. aureus*. A. Structure and (B) light absorption spectrum of HB. (C) Viability of *S. aureus* (5 × 10^7^ CFU ml^‐1^) incubated with varying concentrations (0, 100, 200, 300, 400, 500 nM) of HB and exposed to 460 nm LED light for different light doses (0, 1, 2, 3, 4, 5 J cm^‐2^). The data were represented as log_10_ values of viable bacterial cells of *S. aureus* per ml. (D) Plating images representing the enumeration of viable bacteria with photodynamic treatment. The treatment/dilution conditions corresponding to respective plating locations were indicated in the first line art. E. Bar graphs representing viable bacterial comparisons with additional increment of light fluence to 9 J cm^‐2^. The data were represented as log_10_ values of viable bacterial cells of *S. aureus* per ml. Values represent averages of at least triplicate data, and error bars indicate the standard deviation. *P < 0.05, ***P < 0.001, NS *P* > 0.05.

In consistence with previous findings (Jiang, *et al*., 2013; Jan, *et al*., 2019), HB is a perylene quinone derivative according to its chemical structure (Fig. 1A). Revealed by its absorption spectrum curve, HB has maximum absorption at 460 nm (Fig. 1B); thus, light irradiation with wavelength of 460 nm was used in the following aPDI studies.

To comprehensively investigate its photokilling efficacy against *Staphylococcus aureus*, we measured the bacterial survival rate at varying HB concentrations (0, 100 nM, 200 nM, 300 nM, 400 nM and 500 nM) and light densities (0, 1 J cm^‒2^, 2 J cm^‒2^, 3 J cm^‒2^, 4 J cm^‒2^ and 5 J cm^‒2^). According to the results (Fig. 1C), HB did not exhibit considerable dark toxicity under the tested condition (*P* = 0.291 for ‘500 nM, 0 J cm^‒2^’ vs. sham control), and light irradiation alone did not yield bactericidal effect (*P* = 0.99 for ‘0 nM, 5 J cm^‒2^’ vs. sham control). In contrast, after irradiation with 1 J cm^‒2^, 100 nM HB led to significant reductions in the survival of *S*. *aureus* strain (*P* < 0.05 for ‘100 nM, 1 J cm^‒2^’ vs. ‘0 nM, 1 J cm^‒2^’). The viability was further reduced with increment of light density and HB concentration, suggesting that the HB‐mediated aPDI varied depending on light and HB doses used. When 500 nM HB was irradiated by 5 J cm^‒2^ of blue light, the viable staphylococcal cells were decreased by 4–5 log_10_. The representative plate counting is shown in Fig. 1D. To test if photodynamic inactivation efficiency can be further strengthened, 9 J cm^‒2^ of light intensity was used in the subsequent aPDI trials. Compared to 5 J cm^‒2^, 9 J cm^‒2^ of light irradiation further decreased the survival rate of *S. aureus* (*P* < 0.05 for 100 nM or 500 nM), achieving the maximum killing efficiencies (5–6 log_10_ for 500 nM HB) under the tested conditions (Fig. 1E). Overall, these results demonstrated the robust photokilling effect of HB on *Staphylococcus aureus*.

### aPDI of HB disrupted bacterial membrane integrity

**Fig. 2:**
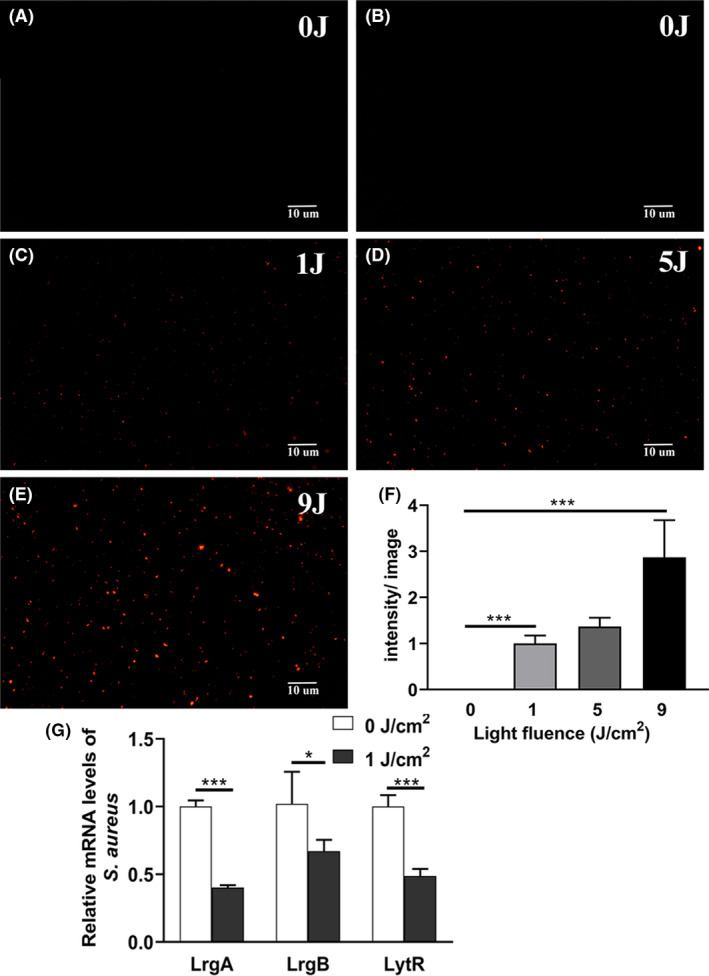
Fluorescence microscopy images of *S. aureus* based on PI staining. A–E. Bacterial cells were treated with HB photoactivated by 460 nm LED light, prior to PI staining, and representative fluorescent images were shown. (A) 0 nM, 0 J cm^‐2^; (B) 500 nM, 0 J cm‐^2^; (C) 500 nM, 1 J cm^‐2^; (D) 500 nM, 5 J cm^‐2^; (E) 500 nM, 9 J cm^‐2^. The graphs were magnified 200 times under microscopy. Scale bars correspond to 10 µm. E. Fluorescence intensities per image for control or treated bacteria, as calculated by ImageJ software. At least 6 separate images for each group were used, and the derived data were normalized against the ‘500 nM, 1 J cm^‐2^’ group. F. Relative mRNA levels of *LrgA*, *LrgB* and *LyrR* in *S. aureus* incubated with 500 nM HB exposed by 1 J cm^‐2^ of light. The expression data of each gene were normalized against the respective dark control. Values represent averages of at least triplicate data, and error bars indicate the standard deviation. ***P < 0.001, *P < 0.05.

We next explored the cellular mechanisms underlying the HB‐mediated aPDI. Since propidium iodide (PI) binds nucleic acids and only enters cells with damaged membranes, it was used here as indicator of membrane integrity of *S. aureus*, an essential factor affecting bacterial survival (Jiang, *et al*., 2013). Based on aPDI results, 500 nM was chosen as lethal dose of HB to conduct this set of trials. As evidenced in Fig. 2A‐F, no apparent loss of membrane integrity was induced by HB in dark condition, and red fluorescence emission remarkedly intensified with the increasing light densities. When 1 J cm^‒2^ of light was applied, fluorescence density started to emerge, and this margin was enlarged to 2.87 ± 0.8 times by 9 J cm^‒2^ of light (*P* < 0.001). These data showed an illumination‐dependent disruption of staphylococcal cell membranes. Of note, as impacted by light dosage, membrane permeability alters in a consistent fashion with bacterial mortality (Fig. 1E), implying a causal‐effect relevance.

To substantiate roles of membrane permeability in the bactericidal effect of aPDI, we further examined the gene expression levels of cell envelope‐related genes, as exemplified by *LrgA/B* and *LytR1/2*, which are responsible for the control of murein hydrolase activity and biofilm formation/maintenance in *Staphylococcus aureus* (Groicher, *et al*., 2000; Lehman, *et al*., 2015). As shown in Fig. 2G, upon the treatment of HB‐mediated aPDI, the expression of *LrgA/B* and *LytR* was downregulated in a significant way (*P* < 0.05). Thus, regulatory system of LrgA/B and LytR was dynamically deregulated by aPDI of HB, supporting the involvement of compromised membrane integrity in the studied decontamination against *Staphylococcus aureus*.

### aPDI of HB altered cell morphology of Staphylococcus aureus

**Fig. 3:**
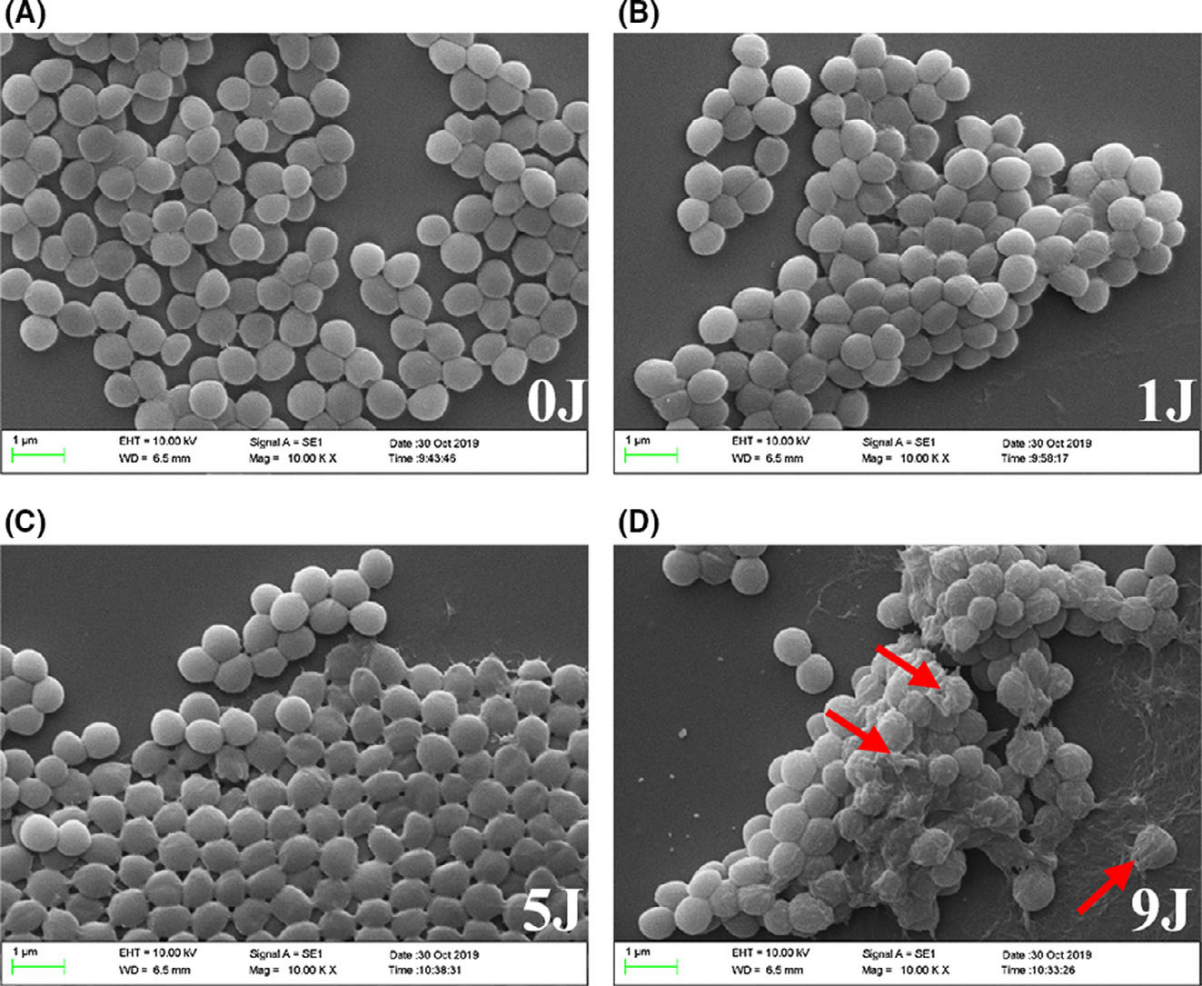
Representative scanning electron microscopy images of *S. aureus* with photodynamic treatment. 500 nM HB was subjected to photoactivation under LED light with a wavelength of 460 nM. The light fluence involved was (A) 0 J cm^‐2^; (B) 1 J cm^‐2^; (C) 5 J cm^‐2^; and (D) 9 J cm^‐2^. Scale bars correspond to 1 µm. Red arrows indicate representative bacterial cells with morphological atrophies. A. Intracellular ROS was analysed using DCFH‐DA staining after HB (500 nM) treatment in combination with light irradiation at a density of 1 J cm^‐2^. Control was designed as HB‐treated sample in dark conditions. Representative fluorescence images are shown in inserts, and scale bar represents 10 µm. With the illumination of 1 J cm^‐2^ or 9 J cm^‐2^, enzymatic activities of SOD (B), CAT (C), as well as extracellular leakage of K^+^ (D) and nucleic acids (E) were shown. Values represent averages of at least triplicate data, and error bars indicate the standard deviation. ***P < 0.001, **P < 0.01, NS *P* > 0.05.

To study the morphological alterations induced by aPDI of HB, staphylococcal cells were subjected to SEM (scanning electron microscope) examination. As revealed by Fig. 3A‐D, while HB alone (500 nM) did not cause any considerable morphological aberrations under dark conditions, light irradiation at a density of 1 J cm^‒2^ altered the cell surface contours of bacteria, and seemingly strengthened the sticky connections between adjacent microorganisms. Cell aggregation became more obvious when the light density was increased to 9 J cm^‒2^. In addition, larger proportion of bacteria displayed wrinkled, irregular surface morphology, with some cellular structures leaked outside of the cell. This indicates that the aPDI‐treated bacteria were damaged, atrophied and lysed, presumably contributing to the increased membrane permeability.

### aPDI of HB damaged staphylococcal cells via oxidative stress

**Fig. 4:**
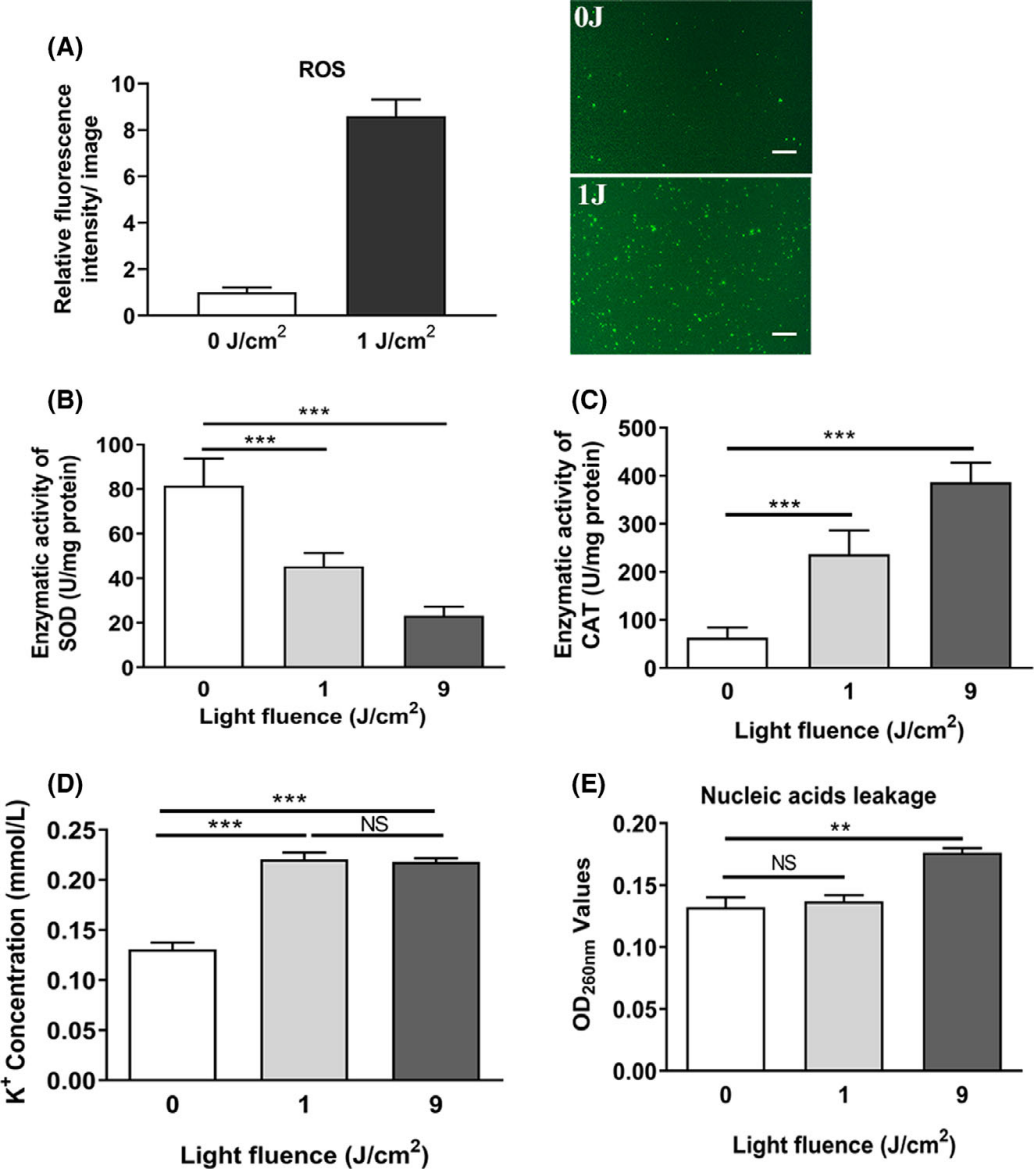
Oxidative stress determination in *S. aureus* with photodynamic treatment. A. Intracellular ROS was analysed using DCFH‐DA staining after HB (500 nM) treatment in combination with light irradiation at a density of 1 J cm^‐2^. Control was designed as HB‐treated sample in dark conditions. Representative fluorescence images are shown in inserts, and scale bar represents 10 µm. With the illumination of 1 J cm^‐2^ or 9 J cm^‐2^, enzymatic activities of SOD (B), CAT (C), as well as extracellular leakage of K^+^ (D) and nucleic acids (E) were shown. Values represent averages of at least triplicate data, and error bars indicate the standard deviation. ***P < 0.001, **P < 0.01, NS *P* > 0.05.

Photodynamic inactivation is known to have bactericidal effects primarily through the generation of intracellular ROS (Lin, *et al*., 2017). To further sort out molecular mechanisms underlying HB‐mediated aPDI, DCFH staining was used to measure ROS levels inside cells of *S. aureus*. As substantial cell death can compromise the accuracy of DCFH staining, only 1 J cm^‒2^ light irradiation was performed. It was shown from Fig. 4A that after treatment with photoactivated HB, the intracellular ROS levels were stimulated to exhibit a significant difference from the basic level (*P* < 0.05), indicating that oxidative stress induced by aPDI of HB was enhanced in the studied context. In terms of antioxidative response of *S. aureus*, the enzymatic activity of SOD (superoxide dismutase) was also curbed upon treatment (*P* < 0.001; Fig. 4B). Interestingly, catalase (CAT) activities displayed an opposite alteration, increasing from (109.67 ± 1.3) U mg^‒1^ to (486.23 ± 15.9) U mg^‒1^ upon exposure of 9 J cm^‒2^ light (Fig. 4C), and this might suggest that bacteria sensed the accumulated hydrogen peroxide and created a response. Overall, unique antioxidative response arose when distinct species of ROS was generated in *S. aureus*.

We next assessed physiological outcome of oxidative stress imposed by aPDI. As a useful indicator to monitor microbial integrity, K^+^ leakage was elevated in the studied circumstances (*P* < 0.001), and no differences were detected between 1 J cm^‒2^ and 9 J cm^‒2^ of light densities (*P* = 0.6; Fig. 4D). Besides, the quantity of extracellular nucleic acids was also increased by 9 J cm^‒2^ irradiation of blue light (*P* < 0.01; Fig. 4E), showing a leakage of bacterial genetic materials. These data suggested that the aPDI‐induced oxidative stress caused the leakage of a variety of intracellular substances in *S. aureus*.

### Curcumin potentiated the photokilling effect of HB on S. aureus

Curcumin was previously identified as an efficient photosensitizer to drive the photodynamic inactivation of microorganisms (Correa, *et al*., 2020). The chemical structure of curcumin used here is presented in Fig. S1A, and its maximum absorption wavelength was measured as 420 nm (Fig. S1B). aPDI activity of curcumin was then assessed against *S. aureus*, and found that upon light irradiation of 1, 5 and 9 J cm^‒2^, curcumin with comparable concentrations with HB did not yield bactericidal capacity (Fig. S2). Due to its proximity to absorption peak of HB (460 nm, Fig. 1B), curcumin was used to supplement the HB‐aPDI regimen to establish a novel dual‐photosensitizer system.

**Fig. 5:**
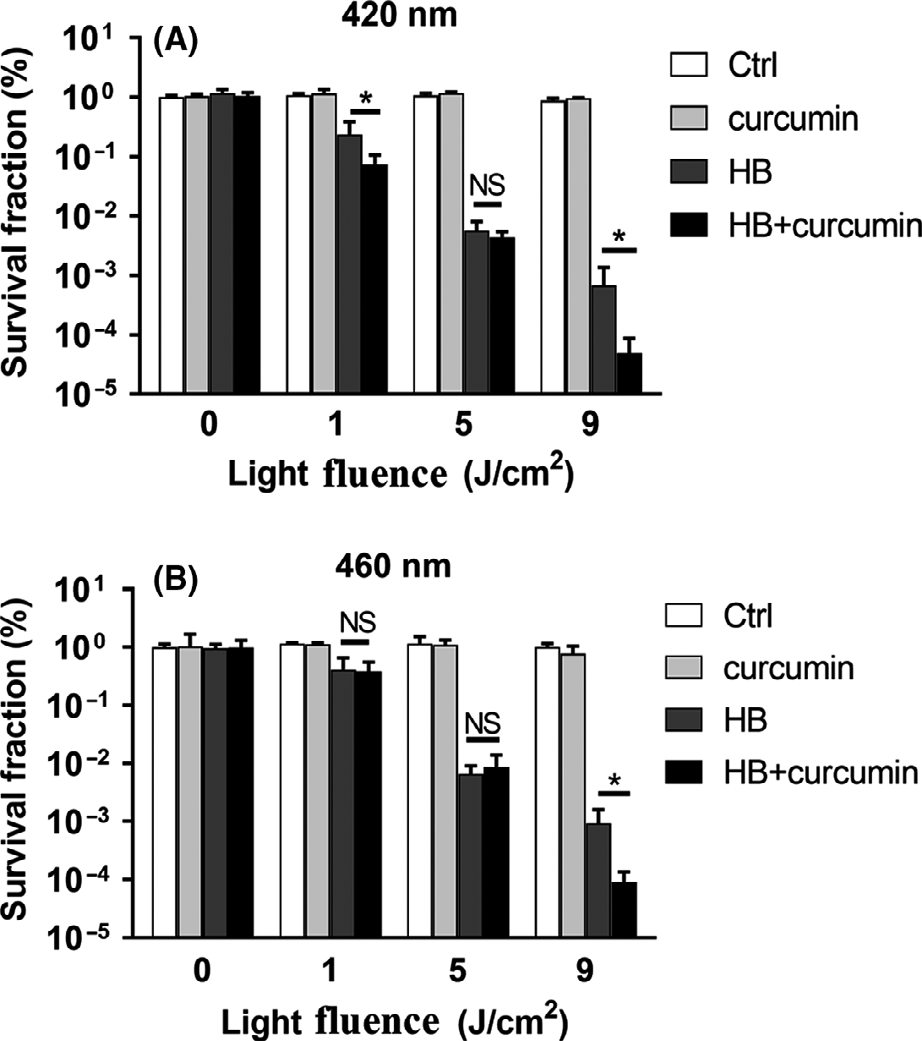
Synergistic effect of curcumin on HB‐mediated photoinactivation of *S. aureus*. Survival fraction of *S. aureus* (5 × 10^7^ CFU ml^‐1^) incubated with 100 nM of HB and/or curcumin exposed to 420 nm (A) or 460 nm (B) LED light for different light doses (0, 1, 5, 9 J cm^‐2^). The survival fraction was calculated based on the percentage of viable cells in comparison to sham control without light irradiation. Values represent averages of at least triplicate data, and error bars indicate the standard deviation. *P < 0.05, NS *P* > 0.05. A–I. Bacterial cells were treated with HB and/or curcumin (100 nM each) photoactivated by 460 nm LED light, prior to PI staining, and representative fluorescent images were shown. A. curcumin, 0 cm^‐2^; (B) HB, 0 J cm^‐2^; (C) HB + curcumin, 0 J cm^‐2^; (D) curcumin, 1 J cm^‐2^; (E) HB, 1 J cm^‐2^; (F) HB + curcumin, 1 J cm^‐2^; (G) curcumin, 9 J cm^‐2^; (H) HB, 9 J cm^‐2^; (I) HB + curcumin, 9 J cm^‐2^. The graphs were magnified 200 times under microscopy. Scale bars correspond to 10 µm. J. Fluorescence intensities per image for control or treated bacteria, as calculated by ImageJ software. At least six separate images for each group were used, and the derived data were normalized against the ‘curcumin, 9 J cm^‐2^’ group. Values represent averages of at least triplicate data, and error bars indicate the standard deviation. *P < 0.05, NS *P* > 0.05.

The concentrations of both photosensitizers were set as 100 nM, under which no significant aPDI activity of curcumin was observed (Fig. 5A and B). When dual photosensitizers were irradiated by 420 nm of light, curcumin was found to potentiate the photokilling effect of HB, with energy density set as either 1 J cm^‒2^ or 9 J cm^‒2^ (*P* < 0.05; Fig. 5A). Since curcumin alone did not display antimicrobial activity, it cooperated with HB to enhance aPDI potency, showing a synergistic effect. This coordination varied depending on the light doses used, whereas 5 J cm^‒2^ irradiation did not show a synergy in this dual system (*P* = 0.446 for HB vs. HB + curcumin). A similar synergistic phenomenon was observed when the imposed light shifted from 420 to 460 nm, a wavelength fit for the release of HB photodynamic activity (Fig. 5B). Under this condition, curcumin still exhibited a robust capacity to enhance the function of HB (*P* < 0.05). However, this synergy only occurred when 9 J cm^‒2^ of irradiation was offered, while no significant potentiation was detected under exposure of 1 J cm^‒2^ (*P* = 0.518 for HB vs. HB + curcumin). This may suggest that a robust illumination was required for 100 nM curcumin to release its synergistic activity at a light wavelength of 460 nm. Besides, the pairwise comparisons between varying aPDI conditions are shown in Table 1.

**Table 1:** The results of pairwise comparisons of *P*‐values in different photodynamic groups (light wavelength = 460 nm).

Groups	Ctrl	Cur‐1J	HB‐1J	Dual‐1J	Cur‐5J	HB‐5J	Dual‐5J	Cur‐9J	HB‐9J	Dual‐9J
Ctrl	–	0.325	<0.05	<0.05	0.493	<0.05	<0.05	0.193	<0.05	<0.05
Cur‐1J	0.325	–	<0.05	<0.05	0.909	<0.05	<0.05	0.191	<0.05	<0.05
HB‐1J	<0.05	<0.05	–	0.874	<0.05	<0.05	<0.05	<0.05	<0.05	<0.05
Dual‐1J	<0.05	<0.05	0.874	–	<0.05	<0.05	<0.05	0.183	<0.05	<0.05
Cur‐5J	0.493	0.909	<0.05	<0.05	–	<0.05	<0.05	0.179	<0.05	<0.05
HB‐5J	<0.05	<0.05	<0.05	<0.05	<0.05	–	0.378	<0.05	<0.05	<0.05
Dual‐5J	<0.05	<0.05	<0.05	<0.05	<0.05	0.378	–	<0.05	<0.05	<0.05
Cur‐9J	0.193	0.191	<0.05	0.183	0.179	<0.05	<0.05	–	<0.05	<0.05
HB‐9J	<0.05	<0.05	<0.05	<0.05	<0.05	<0.05	<0.05	<0.05	–	<**0.05**
Dual‐9J	<0.05	<0.05	<0.05	<0.05	<0.05	<0.05	<0.05	<0.05	<**0.05**	–

Ctrl, sham control group without light irradiation; Cur, curcumin; HB, hypocrellin B; and Dual, HB and curcumin mixture. The concentrations of Cur and HB used were 100 nM each, and the light intensity was denoted with the number per square centimetres. The *P*‐value derived from comparisons of HB‐9J and Dual‐9J was bolded.

### Curcumin aggravated the membrane penetrance caused by aPDI of HB

**Fig. 6:**
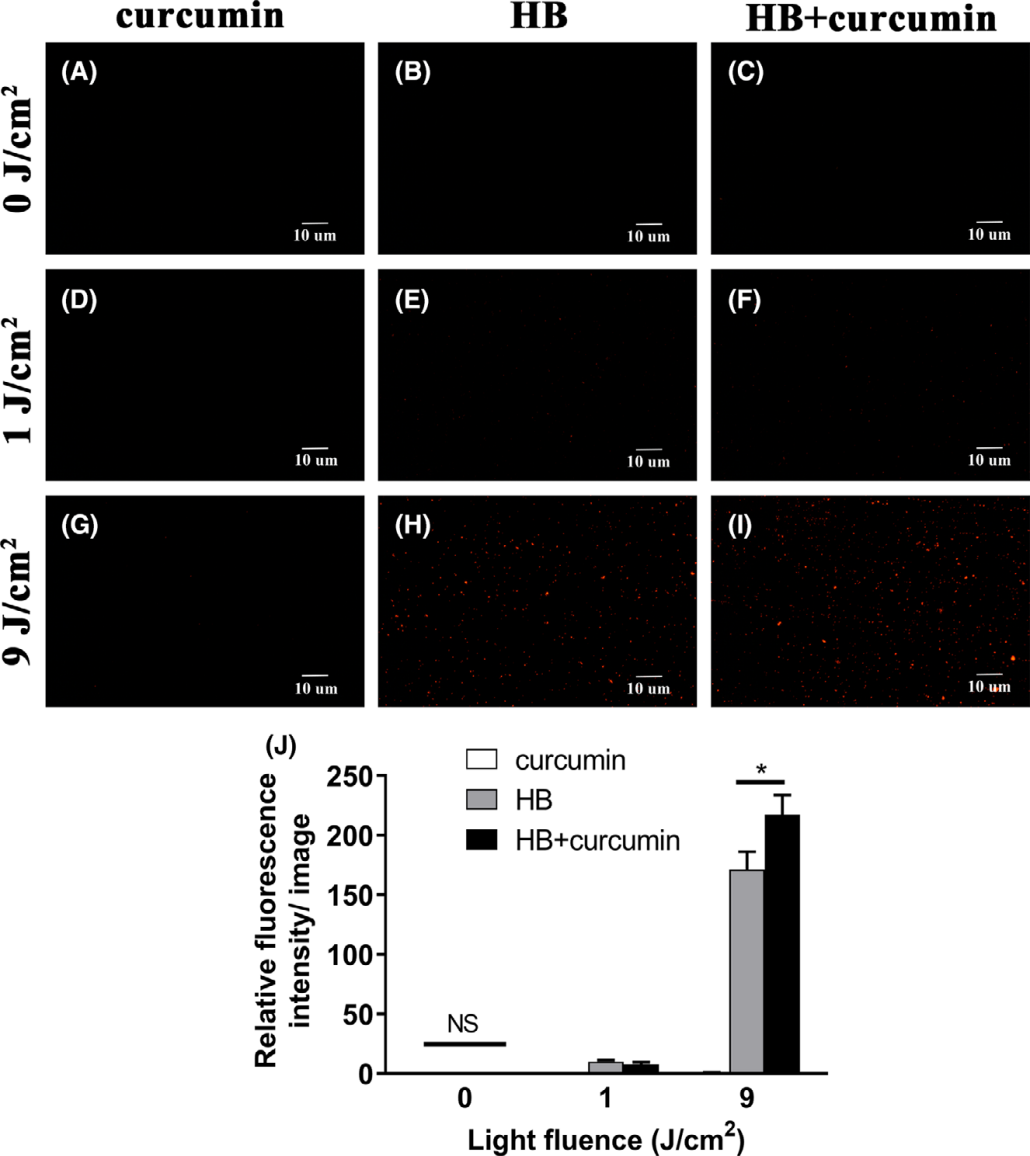
Fluorescence microscopy images of *S. aureus* based on PI staining. A–I. Bacterial cells were treated with HB and/or curcumin (100 nM each) photoactivated by 460 nm LED light, prior to PI staining, and representative fluorescent images were shown. A. curcumin, 0 cm^‐2^; (B) HB, 0 J cm^‐2^; (C) HB + curcumin, 0 J cm^‐2^; (D) curcumin, 1 J cm^‐2^; (E) HB, 1 J cm^‐2^; (F) HB + curcumin, 1 J cm^‐2^; (G) curcumin, 9 J cm^‐2^; (H) HB, 9 J cm^‐2^; (I) HB + curcumin, 9 J cm^‐2^. The graphs were magnified 200 times under microscopy. Scale bars correspond to 10 µm. J. Fluorescence intensities per image for control or treated bacteria, as calculated by ImageJ software. At least six separate images for each group were used, and the derived data were normalized against the ‘curcumin, 9 J cm^‐2^’ group. Values represent averages of at least triplicate data, and error bars indicate the standard deviation. *P < 0.05, NS *P* > 0.05.

Regarding membrane integrity alterations caused by dual photosensitizers (Fig. 6A‐J), no apparent fluorescence was observed under dark environment, indicating no dark toxicity within the studied concentrations. Meanwhile, the amount of PI dyes entering staphylococcal cells were increased by the additional curcumin, from 171.07 ± 15.16 to 216.93 ± 16.74 (normalized data, *P* < 0.05) as irradiated by 9 J cm^‒2^ of 460 nm LED light (Fig. 6A‐J), in comparison with HB‐treated microorganisms alone. Of note, a larger proportion of staphylococcal cells were tightly connected to adjacent cells, forming a variety of microbial aggregates. This phenomenon is consistent with observations from SEM graphs (Fig. 3D). Taken together, membrane integrity of *S. aureus* was further hampered by the additional curcumin‐mediated aPDI.

### aPDI of dual photosensitizers acted by generating diverse species of ROS

**Fig. 7:**
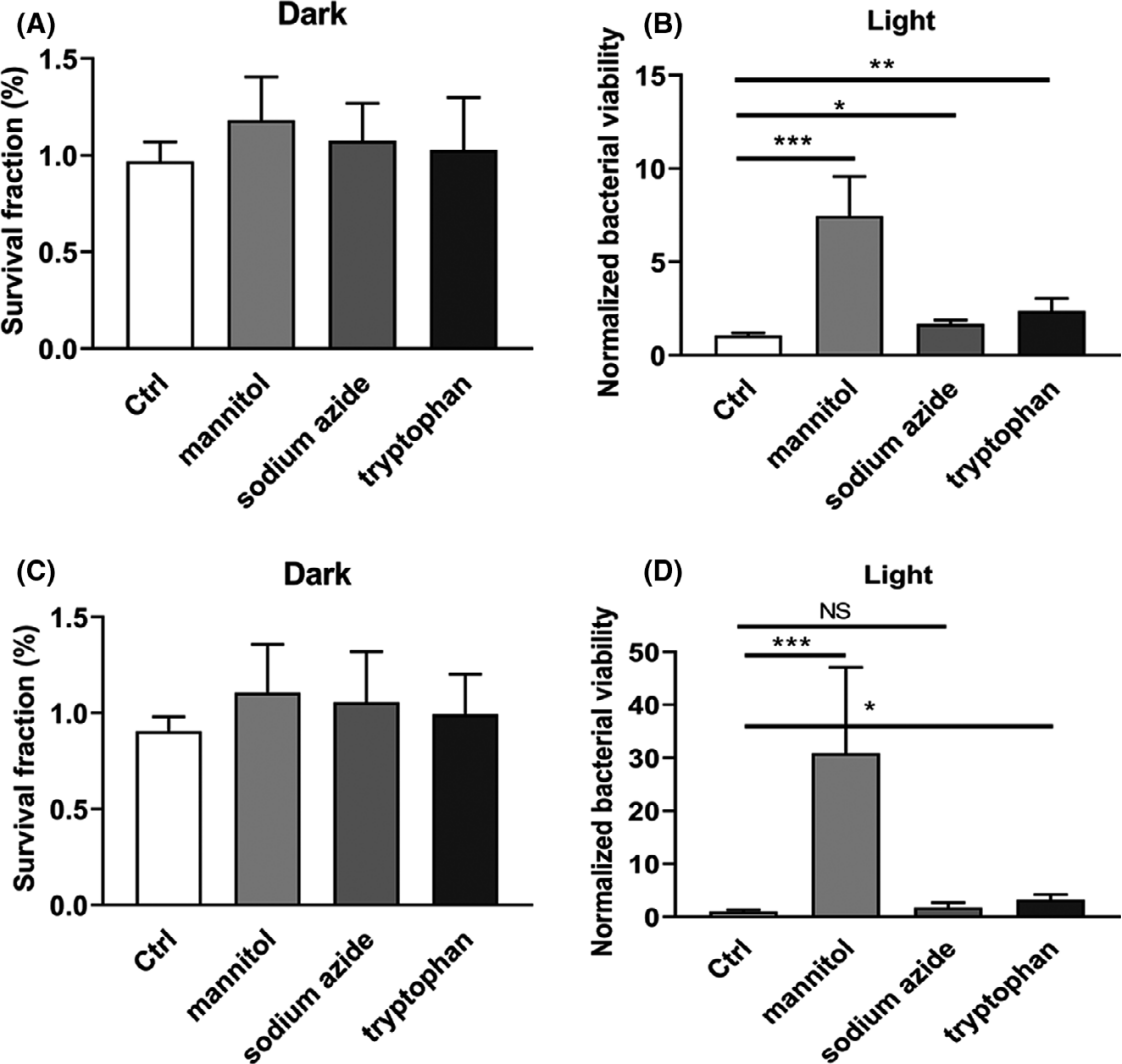
Effect of ROS scavengers and quenchers on the photodynamic inactivation of *S. aureus* by HB. *S. aureus* (5 × 10^7^ CFU ml^‐1^) was incubated with 100 nM of HB (A and B) or 10 µM of curcumin (C and D) exposed to LED light at a fluence of 0 J cm^‐2^ (dark) or 9 J cm^‐2^ (light). A type I scavenger (mannitol, 10 mM), a type II quencher (sodium azide, 1 mM) and a mixed type I‐II quencher (tryptophan, 10 mM) were, respectively, pre‐incubated with the mixture of PS and bacterial suspensions for 30 min prior to illumination. For A and C, the survival fraction was calculated based on the percentage of viable cells in comparison to sham control (PS‐, light‐). For B and D, bacterial viability was normalized against the respective Ctrl group (in the absence of scavenger/quencher) for better visualization and comparisons. Values represent averages of at least triplicate data, and error bars indicate the standard deviation. ***P < 0.001, **P < 0.01, *P < 0.05, NS *P* > 0.05. A. Viable number of *S. aureus* in apple slice treated with 100 nM HB and curcumin photoactivated by 9 J cm^‐2^ of LED light with wavelength of 460 nm. The treated apple was placed at room temperature for 2 h before bacterial enumeration. The data were normalized against the sham control group (S‐L‒). Representative apple slices without dish covers were shown in inserts. B. pH values of apple slices in the presence or absence of photodynamic treatment. C. Total phenol content of apple slices in the presence or absence of photodynamic treatment. S‐L‒, no PS and light; S‐L+, no PS, with light illumination; S + L‒, with PS, no light illumination; S + L+, with PS and light. Values represent averages of at least triplicate data, and error bars indicate the standard deviation. **P < 0.01. D. Schematic representation of dual‐PS‐mediated aPDI against *S. aureus* and its application in apple maintenance.

In order to investigate how curcumin coordinates with HB to strengthen aPDI effect, it is warranted to characterize reaction types of HB and curcumin. Basically, photodynamic reaction can be divided into two distinct types, type I reaction (generating multiple ROS by electron transfer) and type II reaction (generating singlet oxygen by energy transfer) (Kashef and Hamblin, 2017). We use specific scavengers/quenchers like sodium azide (type II), mannitol (type I) and tryptophan (mixed type) to detect the reactive oxygen species generated here. According to Fig. 7 A and B, both sodium azide and mannitol decreased the antimicrobial potency of HB (*P* < 0.05), indicating that HB‐induced reaction can produce both types of ROS. Despite it, HB induced a higher ratio of type I reaction in the studied context, as the bacterial viability was elevated more sharply with the treatment of mannitol relative to sodium azide (Fig. 7B, 7.47 ± 1.81 vs. 1.68 ± 0.19). In the case of 10 µM curcumin (the dosage was chosen on account of its comparable lethal effect under the studied illumination, data not shown), photoactivation at a density of 9 J cm^‒2^ tended to solely induce type I reaction, due that singlet oxygen removal by sodium azide did not improve bacterial survival rate (Fig. 7C and D, *P* = 0.383). Opposed to it, mannitol treatment counteracted the photokilling efficacy of curcumin, as bacterial viability was sharply enhanced (Fig. 7D, *P*< 0.001, 30.9 ± 13.18). Therefore, the individual photosensitizer used here is light‐activated to generate diverse set of ROS combinations, and it is tempting to deduce that the diversity and abundance of type I ROS species, as well as the resulting type I/type II ratio, was increased due to the additional action of curcumin, which might underlie the increased death rate of *S. aureus*. Of note, tryptophan treatment did not show strong potentiation of bacterial survival (Fig. 7B and D), probably resulted from its poor quenching efficiency under the studied circumstance.

### aPDI decontaminated apples from S. aureus while maintaining food quality

**Fig. 8:**
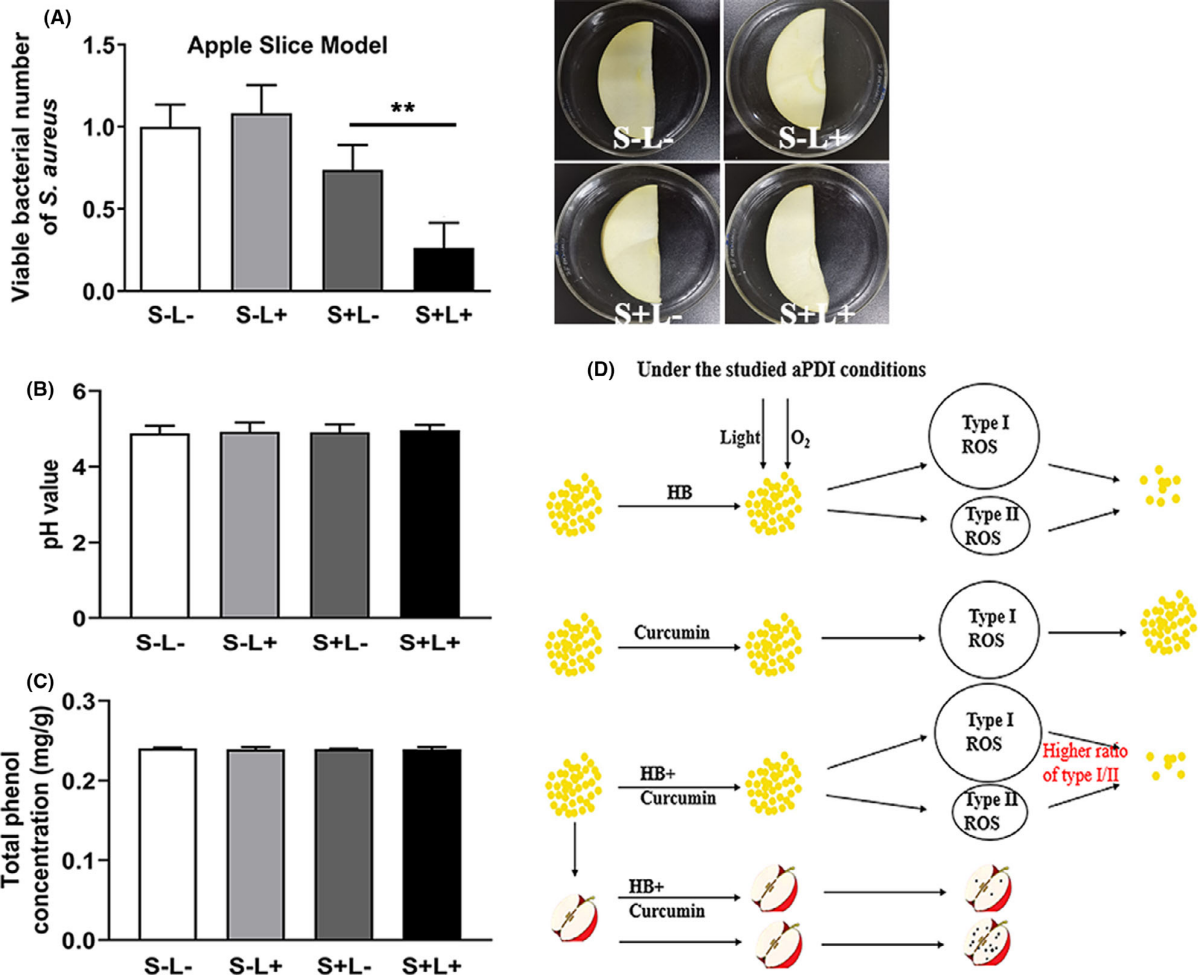
Food decontamination model trails based on dual‐PS‐mediated aPDI. A. Viable number of *S. aureus* in apple slice treated with 100 nM HB and curcumin photoactivated by 9 J cm^‐2^ of LED light with wavelength of 460 nm. The treated apple was placed at room temperature for 2 h before bacterial enumeration. The data were normalized against the sham control group (S‐L‒). Representative apple slices without dish covers were shown in inserts. B. pH values of apple slices in the presence or absence of photodynamic treatment. C. Total phenol content of apple slices in the presence or absence of photodynamic treatment. S‐L‒, no PS and light; S‐L+, no PS, with light illumination; S + L‒, with PS, no light illumination; S + L+, with PS and light. Values represent averages of at least triplicate data, and error bars indicate the standard deviation. **P < 0.01. D. Schematic representation of dual‐PS‐mediated aPDI against *S. aureus* and its application in apple maintenance.

Photokilling efficacy of HB and curcumin against *S. aureus* was then tested in an apple contamination model. When fresh apples were inoculated with *S. aureus*, half of samples were subjected to light irradiation based on dual photosensitizers. It is then revealed that, compared to apples under dark environment, the number of this food‐derived microorganisms significantly declined on apples receiving visible light irradiation during the storage of 2 h (Fig. 8A *, P *< 0.01). This indicates that the aPDI used here is potent in *bona fide* removing *S. aureus* from representative food products. Besides, under the tested conditions, HB alone can also reduce *S. aureus* contamination with the visible light, while curcumin failed to yield bactericidal effect (Fig. S3A).

Subsequently, influence of aPDI treatment on apple quality was assessed. As seen from Fig. 8B and Fig. S3B, this antimicrobial treatment did not significantly change the pH value of the tested apples. Meanwhile, total phenolic content also did not show significant differences between treatment (Fig. 8C, Fig. S3C). These results might suggest that apple quality was not affected by the HB/curcumin‐mediated aPDI.

## Discussion

Health concerns raised by foodborne pathogens such as *Staphylococcus aureus* have increased in recent years, but efforts to control it were often circumvented due to the loss of antimicrobial agent efficacy and slow development of new antibiotics or approaches (Crago, *et al*., 2012; Marquez, *et al*., 2019; Yu, *et al*., 2019). Introducing photodynamic therapy (PDT) to antimicrobial application towards food‐related bacteria, namely aPDI, was a new and rapidly evolving solution. aPDI of HB and curcumin has several considerations to be merited: natural source, non‐toxic to human, strong light adsorption in the visible region and high photostability (Xu, *et al*., 2002; Gao, *et al*., 2019). This aPDI technique could be viewed as an optimal alternative for UV light usage in food preservation, which is harmful to human and causes decolorization in certain products at high doses (Kim, *et al*., 2017). Of note, it remains questionable if bacteria can develop resistance against aPDI, but a previous attempt to model aPDI resistance found that, after repeated exposure to aPDI 10 times, no resistance was developed for *Escherichia coli* or *Vibrio fischeri* (Tavares, *et al*., 2010, Kashef and Hamblin, 2017). Therefore, aPDI is a promising method to address foodborne microorganisms with antibiotic resistance. Given these advantages, a better understanding of modes of action of aPDI is required, whereas a synergistic effect of hypocrellin B and curcumin was here proposed and studied in detail.

Hypocrellin B is known to elicit photodynamic reaction. Regarding *S. aureus,* HB displayed a strong bactericidal activity against this prevalent foodborne pathogen (Fig. 1C). When 1 J cm^‒2^ of blue light was provided, a minimum of 100 nM HB was sufficient to yield lethal effect. And to gain the similar consequence, at least 10 µM curcumin should be used (data not shown). This proposes HB as a potent candidate compound to drive the photodynamic inactivation of *S. aureus*. In other aspects, the derived antimicrobial efficiency is difficult to be compared with a previous report (Jiang, *et al*., 2014), which saw the staphylococcal survival rate drop 1–2 log_10_, as treated with 2.5 uM HB and 0.3 J cm^‒2^ light. In this study, we profiled photokilling characteristics of HB by 5 intervals of PS dosages (100, 200, 300, 400 and 500 nM) and 6 intervals of light dosages (1, 2, 3, 4, 5, 9 J cm^‒2^). The reductions of bacterial viability were 1–6 log_10_; thus, the increased light density may play profound roles in controlling antimicrobial efficiency, as it can drive the relatively smaller dose of HB (than 2.5 µM as referenced) to inhibit the growth of *S. aureus*.

It was proposed that ROS generated by light‐activated PS can damage microbial cells via three mechanisms: (i) damage of cell membrane; (ii) inactivation of essential enzymes; and (iii) damage to DNA (Hamblin, 2016). In the case of HB, cell membrane integrity was severely damaged owing to light activation (Fig. 2), allowing the fluorescent dyes to enter cells and escape from exclusion pathways. This mode of action is not difficult to anticipate, due that visible light primarily converted energy into PS embedded in cell envelope. The reaction enables cell surface structure a prime site to be exposed by ROS. Further studies are needed to unveil the dynamic locations of HB on *S. aureus* to test this argument, which is partially supported by deregulation of cell envelope‐related genes, like *LrgA/B* and *LytR*.

In terms of essential enzymes, aPDI treatment hampered the enzymatic activity of superoxide dismutase, while stimulating catalase action (Fig. 4B and C). This might reflect a divergent capacity of microorganisms, in the studied context, to sense and respond to distinct species of reactive oxygen. In light of its aerobic nature, *Staphylococcus aureus* harbours complicated defence systems in response to oxidative stress (Gaupp, *et al*., 2012). However, since HB can generate singlet oxygen via type II reaction, it is highly unlikely for *S. aureus* to develop resistance towards this new form of ROS, which may, in turn, contribute to the impaired membranes and the sterilized cells. Besides, a stimulated catalase was not detected in another aPDI process based on 5,10,15,20‐tetrakis(1‐methylpyridinium‐4‐yl)porphyrin tetraiodide, which did not change catalase activity of *S. aureus* (Bartolomeu, *et al*., 2016). Therefore, it can speculate that distinct set of ROS generated and specific microbial response are decided by the given PS and its microenvironment.

PS was routinely used with other antimicrobials or approaches to enhance its photodynamic potency. For instance, antibiotics can be used as aPDI adjuvants, giving rise to synergistic action (Wozniak, *et al*., 2019); UV coordinated with aPDI to release its antimicrobial activity (Correa, *et al*., 2020). Amounting attempts were made to integrate the use of nanoparticles with aPDI, to improve PS delivery to microorganisms, increase the singlet oxygen yields, sometimes to act as PS themselves like TiO_2_ and quantum dots (Kashef and Hamblin, 2017). Combinatorial use with HB can extend the photoresponse of TiO_2_ to visible light and achieve high ROS yields under visible light illumination. In light of it, new strategies were introduced to mix two PSs to gain a robust system for photodynamic eradication of microorganisms. The combinations of PS often resulted in conflicting aPDI outcome, as equivalent mixture of curcumin and CXE (*Curcuma xanthorrhiza* extract) substantially reduced the viability of *S. mutans* (Lee, *et al*., 2017), whereas two‐photon system based on HA and HB showed similar potency with one‐photon photodynamic process (Liu, *et al*., 2002). The present investigation revealed a synergistic antimicrobial effect induced by a low dosage and equivalent mixture of HB and curcumin (Fig. 5A and B). HB and curcumin were predisposed to act together based on their similar wavelength of excited light (420 nm for curcumin, 460 nm for hypocrellin B). That is, when one photosensitizer is photoactivated by its excited light, the same illumination is likely to activate the other photosensitizer simultaneously, generating a strengthened bactericidal potency. This theory was then tested, and as a result, the established dual‐photon system was conditionally potent in killing *S. aureus*, showing, to an extent, collaboration of the studied PSs. Therefore, aPDI is assumed to be selective in inactivation and eradication of specific microorganism, and efficacy induced by different set of PSs is highly trial and error, relying on the nature of PSs and their treatment conditions.

One intriguing discovery is that, in the studied context (100 nM, 1–9 J cm^‒2^), light‐activated curcumin alone did not cause considerable deaths of *S. aureus* (Fig. S2). And when it was supplemented into aPDI regimen of HB, the photodynamic activity was strongly potentiated (Fig. 5). That might suggest that, although no obvious reductions of bacterial viability were measured, the sole treatment of 100 nM curcumin has also exerted some form of adverse effect on the microbial structure and/or its physiology. This sublethal dose of curcumin may predispose microorganisms to a more susceptible status to a routine PDI mediated by HB. This argument was supported by a previous statement that aPDI sensitized *Acinetobacter baumannii* and enhanced its prowess to antimicrobials (Wozniak, *et al*., 2019). Of interest, however, under 1 J cm^‒2^ illumination, the synergy merely occurred when 420 nm wavelength of light was provided, whereas 460 nm did not lead to the comparable results. As the maximum absorption wavelength of curcumin was 420 nm, these data may suggest that the sublethal dose of curcumin (100 nM in this study) can only sensitize *S. aureus* in its best light‐activated state. However, it remains questionable if this strict light selectivity is consistent when concentration of curcumin is elevated to a proximity of lethal levels (10 µM, for instance). A reference case is offered by the increased light intensity: when light irradiation was robust enough (9 J cm^‒2^), a wavelength shift from 420 to 460 nm can still witness the synergistic roles of curcumin in HB‐mediated aPDI (Fig. 5B and Fig. 6). Still, strong irradiation did not always correspond to a better chance to induce a synergistic aPDI, as evidenced by the poor performance of 5 J cm^‒2^ (compared to 1 J cm^‒2^) under both conditions. As a conclusion, synergy of HB/curcumin‐mediated aPDI is highly dependent on the photodynamic conditions used.

Although the *bona fide* mechanisms underlying aPDI synergy are not yet identified, some alternative hypotheses can be currently drawn (Wozniak and Grinholc, 2018). One possible mechanism could be the contradictive cellular response of *S. aureus* prompted by distinct photodynamic activity of HB and curcumin, which, upon the addition of curcumin, inhibited the expression of genes governing microbial antioxidative stress. This theory can also easily explain the variability of synergy under distinct aPDI conditions, because intensity and orientation of bacterial response are highly variable due to specific extracellular signals. Another possible mechanism is that the added complexity of oxidative stress acts beyond the limits that microorganisms can cope with. The aPDI performance treated with variable quenchers seemed to support this hypothesis (Fig. 7). This trial reveals that HB prompted both type I and type II reactions, but curcumin was solely associated with production of type I ROS, indicating that the individual PS has its characteristic photodynamic preference. Then, the dual‐photon system may generate a unique combination of ROS (especially an enlarged I/II ratio in this case), which, instead of total amounts, promotes the death of a specified bacteria (Fig. 8D). To further test this hypothesis, the detailed reactive oxygen species involved in the studied aPDI are warranted to be dissected in future investigations.

Due to natural origin of hypocrellin B and curcumin, it makes sense to take advantage of this aPDI approach to food application. In the example of apple decontamination presented here (Fig. 8), up to 74 % of viability of *S. aureus* was reduced by light‐activated HB/curcumin. This effect was achieved by a relatively low dose of PS (100 nM), whereas no bactericidal effect was detected for curcumin alone in this condition (Fig. S3A). The data can be compared with the consequences of large dose of curcumin, with greatest reduction of (2.0 ± 0.4) log_10_ achieved by 80 µM and 10 J cm^‒2^ (Correa, *et al*., 2020). Besides, although it is long considered to have antimicrobial activity *per se*, curcumin (Silva, *et al*., 2017; Hernandez‐Patlan, *et al*., 2018), restrained by the studied concentration, did not show remarkable inhibitory effect on bacterial survival in this study (Fig. S2). As an alternative for UV treatment, massive merits are provided by use of visible light, and of utmost importance, the sterilization process is liberated from spatiotemporal restrictions, continuing to kill food‐derived bacteria and maintain food quality. Therefore, although *bona fide* storage conditions are different from laboratory models, such as small‐scale microbial invasion, long‐term illumination of natural light, shelf life for a variety of food forms is anticipated to be prolonged and the relevant specifications are currently under study. Besides, it is noteworthy that this aPDI was mainly directed towards the superficial microorganisms of fresh apples, owing to the low penetrability of visible light used.

In conclusion, hypocrellin B‐mediated aPDI was found potent in eradiation of foodborne pathogen, *Staphylococcus aureus*. The bactericidal efficacy was achieved by disrupting cell envelope integrity, as well as by deregulating the microbial antioxidative mechanisms. Curcumin strongly potentiated the photokilling capacity of HB, forming an effective aPDI system mediated by dual photons, which was successfully applied in apple decontamination while maintaining food quality. This study shows that photodynamic inactivation based on dual photons could serve as a new and promising approach to control food‐related bacteria.

## Experimental procedures

### Bacterial strains and growth conditions

*Staphylococcus aureus* ATCC25923 was purchased from BeNa Culture Collection (Shanghai, China). Microbial strains were cultured in brain heart infusion broth (Gibco; Thermo Fisher, Beijing, China) and grown at 37°C in a shaker at 200 rpm. Exponential bacterial culture was obtained by growing *S. aureus* in fresh medium for approximately 1 h to a density of 10^7^–10^8^ colony‐forming units (CFU) ml^‐1^, with optical density (OD) value at 600 nm reaching 0.5–0.6. Approximately 5 × 10^7^ cells were collected at 5000 rpm for 5 min, resuspended in 1 ml of PBS (phosphate‐buffered saline) and then subjected to the following photodynamic trials.

### Photosensitizers and light source

Hypocrellin B and curcumin were purchased from Yuanye Biotech (Shanghai, China). HB and curcumin were dissolved in dimethylsulphoxide (DMSO) at a concentration of 1 M and 7.5 mM respectively. Solution passed through a 0.22 um‐pore‐size filter for sterilization and stored at 4°C in the dark till use. The absorption spectrum of each photosensitizer was recorded at room temperature with a Microplate Spectrophotometer (Infinite 200 Pro; Shanghai, China). LED light source with wavelength of 420 nm or 460 nm (CREE; Durham, NC, USA) was used to deliver light to wells in 24‐well plates. The power was adjusted to 41.2 mW cm^‒2^ or 50.8 mW cm^‒2^ for 420 or 460 nm LED devices respectively. A laser power meter (VLP‐2000; Beijing, China) was used to measure the light output power, and the above values were calibrated prior to irradiation experiments. The ordinary chemicals and reagents were of analytical grade and obtained from Sinopharm (Beijing, China).

### aPDI treatment

aPDI treatment was performed as previously described (Demidova and Hamblin, 2005), with some modifications: approximately 5 × 10^7^ CFU ml^‒1^ bacteria was incubated with varying concentrations of photosensitizers (0–900 nM for HB; 0–500 nM, 10 µM for curcumin; 0 and 100 nM each for dual photon) under dark environment for 1 h. Then, aliquots of 1 ml were placed in 24‐well plates and illuminated with the appropriate light at room temperature. Fluences ranged from 0 to 9 J cm^‒2^ at a power density of 41.2 mW cm^‒1^ or 50.8 mW cm^‒1^. All aPDI experiments were compared with three independent control groups: sham control (light‐, PS‐), PS alone (light‐, PS+) and light alone (light+, PS‐). For dual‐photo trials, 100 nM HB and curcumin were mixed prior to incubation with *S. aureus* strains. For enumeration of viable cells, 10‐fold serial dilutions were prepared in sterile PBS (10^‐1^ to 10^‐5^) from each treated and control samples. Aliquots of 100 µl were plated in beef extract peptone medium (0.3% beef extract, 1% peptone, 0.5% NaCl, 1.2–1.5 % agar powder). The plates were incubated at 37°C for 48 h, and the number of colonies was counted. Besides, aliquots of 1.5 µl were pipetted onto the appropriate position of plates for multiple colonial observation. The survival fraction was calculated relative to sham control (light‐, PS‐). All experiments were carried out in triplicate.

### Bacterial membrane permeability detection

Propidium iodide (PI) was used to detect bacterial membrane permeability. Following aPDI treatment, bacterial cells were harvested by centrifugation at 5000 rpm for 5 min at 4°C and washed twice with sterile PBS buffer. Bacterial cells were then incubated with PI at 37°C under dark environment for 15 min. After washed with PBS buffer, cells were transferred to a cover glass slide by pipette and observed using fluorescence microscopy (Nikon; Tokyo, Japan). The images were recorded at the excitation wavelength of 460–550 nm and emission wavelength of 590 nm. Red fluorescence intensity per image was measured from ImageJ software (NIH, Bethesda, MD, USA) and then subjected to statistical comparison.

### qPCR analysis

The total RNAs were extracted using the RNeasy Protect Bacteria Mini Kit (Qiagen, Shanghai, China) from *S. aureus* at exponential phase (OD_600nm_ = 0.5–0.6). Subsequently, random 6 mers were used to complete the reverse transcription reaction, according to the manufacturer’s instructions (PrimeScript RT Master Mix, Takara; Dalian, China), resulting in the first stands of total cDNA. qPCR was performed based on cDNA templates using the Roche LightCycler 96 (Shanghai, China). The primers used are listed in Table 2. The relative expression of individual gene was quantified using 16S rRNA as internal control.

**Table 2:** Primers used in this study.

Primers	Sequences (5’‐3’)	Gene
LrgAF	ACGCATCAAAACCAGCACA	*LrgA*
LrgAR	CGCCTAACTTAACAGCACCAG	
LrgBF	CACCGCTATTTGTCAGTATGG	*LrgB*
LrgBR	CAATACCTCCGATGATACGATG	
LytRF	AAGAGCCACCTGCGATTA	*LytR*
LytRR	GCATCTTTGGCTTTAGTCG	
27F	AGAGTTTGATCCTGGCTCA	*16S rRNA*
1492R	GGTTACCTTGTTACGACTT	

### Intracellular ROS measurement

After photodynamic treatment, *S. aureus* strains were incubated with 10 μM DCFH‐DA (2’, 7’‐dichlorofluorescein diacetate) fluorescent dye (Sigma‐Aldrich, Shanghai, China) for 30 min at room temperature. The fluorescent signals were observed from fluorescent microscopy (Nikon) excited at 485 nm, and intracellular ROS levels were calculated through fluorescent quantification using ImageJ software.

### Scanning electron microscopy (SEM)

To assess the alteration of *S. aureus* membrane morphology and ultrastructure, SEM was performed as described previously (Jan, *et al*., 2019). The bacterial suspension was transferred on glass coverslips and fixed in fresh 2.5 % glutaraldehyde at 4°C overnight. Subsequently, the coverslips were washed with PBS and incubated with 1 % osmium tetroxide at 4°C for 2 h. Following ethanol dehydration, the samples were freeze‐dried, coated with gold and observed using a SEM (X650, Hitachi; Tokyo, Japan).

### Measurement of intracellular potassium and DNA leakage

Following aPDI treatment, 1 ml culture from treated or control groups was subjected to centrifugation at 5000 rpm for 5 min, and the supernatant was collected for further examination. Potassium leakage was measured using K^+^ assay kit (Jiancheng; Nanjing, China), according to the manufacturer’s instructions. DNA concentration was determined according to UV absorption at a wavelength of 260 nm, using UV‐2600 spectrophotometer (Shimadzu; Kyoto, Japan).

### Enzymatic activity assay

Following aPDI treatment, bacteria from varying groups were harvested by centrifugation at 5000 rpm for 5 min and then resuspended in sterile PBS buffer with equivalent volume. Enzymatic activities of SOD and CAT were determined with SOD assay kit (Jiancheng; Nanjing, China) and CAT assay kit (Jiancheng), respectively, according to the manufacturers’ instructions.

### Reaction type determination of photosensitizers

In order to figure out the photodynamic reaction mechanisms associated with respective PS, appropriate ROS scavengers/quenchers were used. A type I scavenger (mannitol, 10 mM), a type II quencher (sodium azide, 1mM) and a mixed type I‐II quencher (tryptophan, 10 mM) were, respectively, pre‐incubated with the mixture of PS (100 nM HB or 10 µM curcumin) and bacterial suspensions for 30 min prior to illumination. Following aPDI treatment at a fluence of 9 J cm^‐2^, bacterial survival rate was calculated and compared through diluted plate counting as described above.

### Photodynamic treatment on fresh apples

Fresh Fuji apples were purchased from a local supermarket in Hefei and stored at 4°C. Apples were washed three times and dried before being cut into approximately 10 g slices in a semicircle shape (60 mm diameter, 8–10 mm thickness) in an aseptic cabinet. For inoculation with the microorganisms, the fresh‐cut apples were soaked in *S. aureus* suspension (10^8^ CFU ml^‐1^) and were kept in an aseptic chamber for 30 min for continuous inoculation. For aPDI assay, samples were incubated with dual PS with individual concentration of 100 nM for 30 min at room temperature in the dark. After being dried for 10 min, apple slices were placed in sterile Petri dishes in the absence of covers, subjected to 460 nm LED illumination of 9 J cm^‐2^ and then incubated at room temperature for additional 2 h in the natural light environment. The non‐illuminated and/or non‐PS‐treated apples were used as controlling groups for comparisons. The controlling apples were also incubated for another 2 h in the natural light environment before homogenesis. The treated fruits were taken, transferred in 10 ml sterile PBS and homogenized. Appropriate dilutions of sample suspension were plated on beef extract peptone medium and kept in the incubator for 24 h at 37°C. The number of colonies was then enumerated manually to assess the aPDI efficacy.

### Measurement of product quality

Following aPDI treatment, food quality of apple slices was further examined. The pH values were measured with a pH meter (Lei‐ci, Shanghai, China). The pH meter was pre‐calibrated using standard solutions before use. Total phenolic content was determined by the Folin–Ciocalteu method (Park and Ha, 2019). Briefly, 0.1 ml of apple homogenates were mixed with 5 ml of 0.2 N Folin–Ciocalteu reagent. Following the additional incubation at room temperature for 3 min, 4 ml of 7.5% sodium carbonate was added and the mixture was shaken and stored for 2 h in the dark. Subsequently, absorbance was measured at 765 nm using a spectrophotometer. Total phenolic contents were expressed as gallic acid equivalents (g kg^‐1^) on a fresh weight basis.

### Statistical analysis

All experiments were performed in at least triplicate samples or plates. Graph data were expressed by mean ± SD (standard deviation). Statistical analysis was performed using SPSS software (version 19.0, IBM; NY, USA). Independent‐sample t‐test was used to indicate two group comparisons, and analysis of variance (ANOVA) with post hoc test was used to perform multiple (>2) comparisons. A *P*‐value lower than 0.05 indicated a significant difference.

## Conflict of interest

None declared.

## Supporting information


**Fig. S1**. Chemical structure (A) and light absorption spectrum (B) of curcumin used in this study. The absorption peak arises in the proximity of 420 nm of wavelength

**Fig. S2**. Survival fraction of *S. aureus* (5 × 10^7^ CFU ml^‐1^) incubated with varying concentrations (0, 100, 200, 300, 400, 500 nM) of curcumin exposed to 420 nm LED light for different light doses (0, 1, 5, 9 J cm^‐2^). The survival fraction was calculated based on the percentage of viable cells in comparison to sham control without light irradiation. Values represent averages of at least triplicate data and error bars indicate the standard deviation

**Fig. S3**. Food decontamination model trails based on individual PS‐mediated aPDI. (A) Viable number of *S. aureus* in apple slice treated with 100 nM HB or 100 nM curcumin photoactivated by 9 J cm^‐2^ of LED light with wavelength of 460 nm. The treated apple was placed at room temperature for 2 h before bacterial enumeration. The data was normalized against the sham control group (S‐L‒). (B) pH values of apple slices in the presence or absence of photodynamic treatment. (C) Total phenol content of apple slices in the presence or absence of photodynamic treatment. S‐L‒, no PS and light; S‐L+, no PS, with light illumination; S + L‒, with PS, no light illumination; S + L+, with PS and light. Values represent averages of at least triplicate data and error bars indicate the standard deviation. ***P < 0.001.
