# Supplementary figures and images for: Synergistic effect of hypocrellin B and curcumin on photodynamic inactivation of Staphylococcus aureus
Source: Microb Biotechnol. 2021 Jan 19;14(2):692–707. doi: 10.1111/1751-7915.13734 (PMC7936292; doi:10.1111/1751-7915.13734)

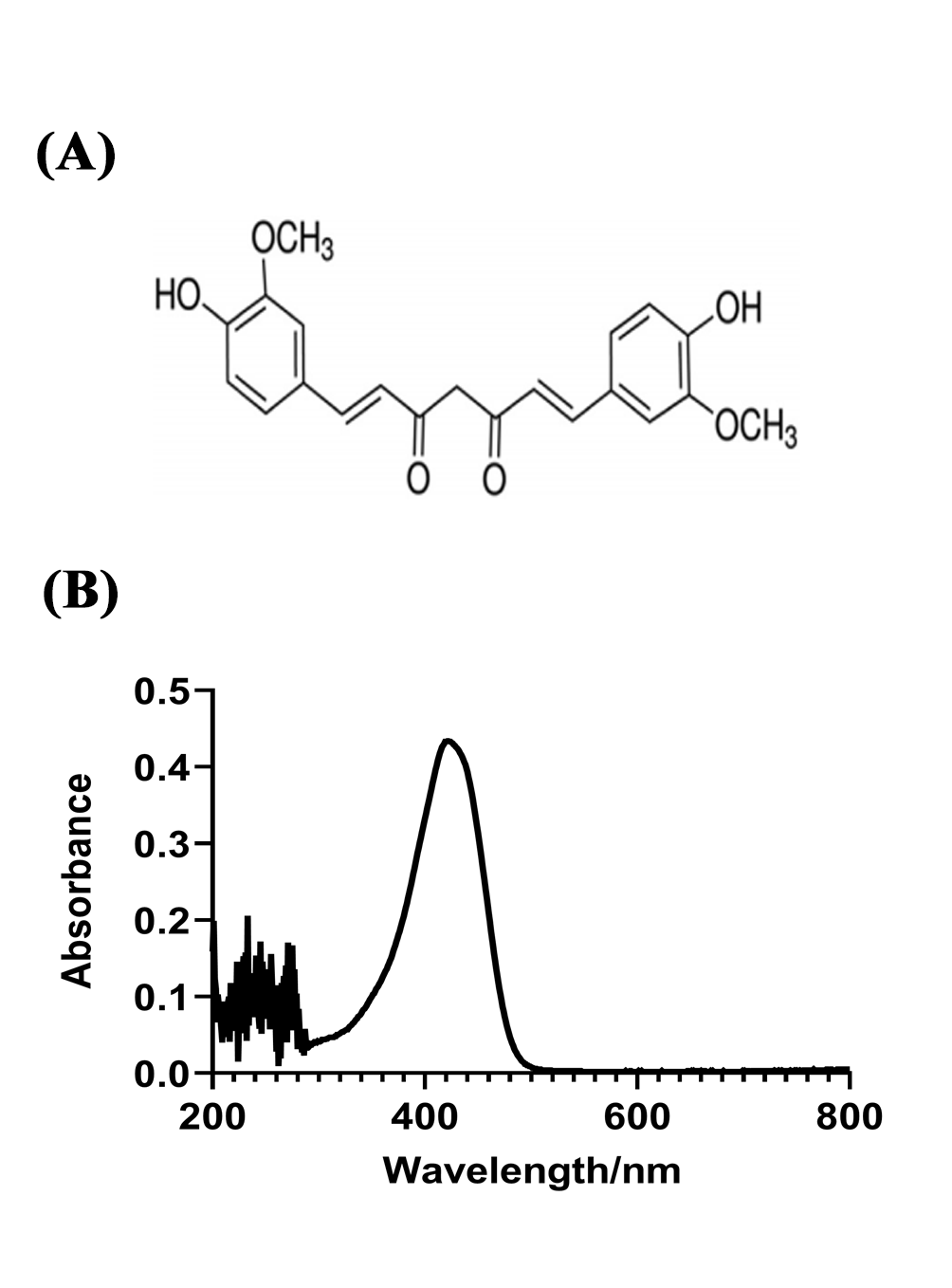

Supplement: Supplementary file 1 — Fig. S1. Chemical structure (A) and light absorption spectrum (B) of curcumin used in this study. The absorption peak arises in the proximity of 420 nm of wavelength [file MBT2-14-692-s001.tif]

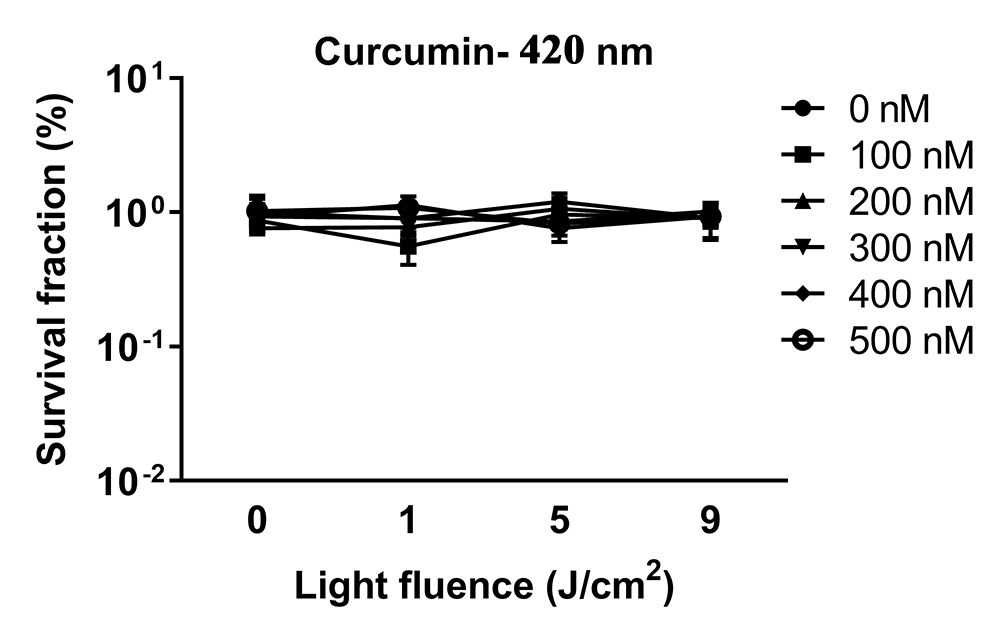

Supplement: Supplementary file 2 — Fig. S2. Survival fraction of S. aureus (5 × 10^7^ CFU ml‐1) incubated with varying concentrations (0, 100, 200, 300, 400, 500 nM) of curcumin exposed to 420 nm LED light for different light doses (0, 1, 5, 9 J cm‐2). The survival fraction was calculated based on the percentage of viable cells in comparison to sham control without light irradiation. Values represent averages of at least triplicate data and error bars indicate the standard deviation [file MBT2-14-692-s002.tif]

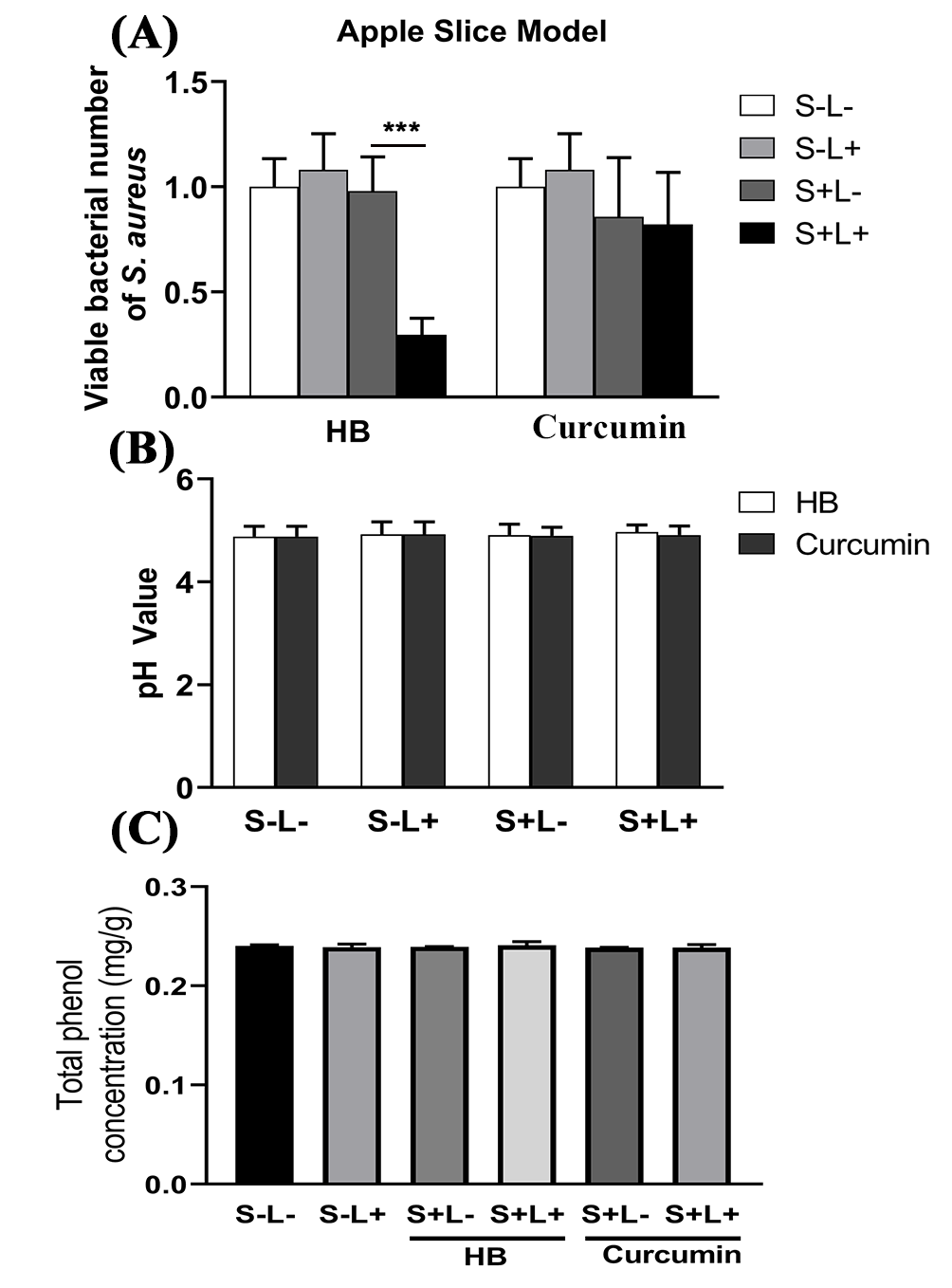

Supplement: Supplementary file 3 — Fig. S3. Food decontamination model trails based on individual PS‐mediated aPDI. (A) Viable number of S. aureus in apple slice treated with 100 nM HB or 100 nM curcumin photoactivated by 9 J cm‐2 of LED light with wavelength of 460 nm. The treated apple was placed at room temperature for 2 h before bacterial enumeration. The data was normalized against the sham control group (S‐L‒). (B) pH values of apple slices in the presence or absence of photodynamic treatment. (C) Total phenol content of apple slices in the presence or absence of photodynamic treatment. S‐L‒, no PS and light; S‐L+, no PS, with light illumination; S + L‒, with PS, no light illumination; S + L+, with PS and light. Values represent averages of at least triplicate data and error bars indicate the standard deviation. ***P < 0.001. [file MBT2-14-692-s003.tif]
